# Influence of Resistance Training Proximity-to-Failure on Skeletal Muscle Hypertrophy: A Systematic Review with Meta-analysis

**DOI:** 10.1007/s40279-022-01784-y

**Published:** 2022-11-05

**Authors:** Martin C. Refalo, Eric R. Helms, Eric. T. Trexler, D. Lee Hamilton, Jackson J. Fyfe

**Affiliations:** 1 Centre for Sport Research (CSR), School of Exercise and Nutrition Sciences, Deakin University, Geelong, VIC Australia; 2 Sport Performance Research Institute New Zealand (SPRINZ), Auckland University of Technology, Auckland, New Zealand; 3 Trexler Fitness LLC, Raleigh, NC USA; 4 Institute for Physical Activity and Nutrition (IPAN), School of Exercise and Nutrition Sciences, Deakin University, Geelong, VIC Australia

## Abstract

**Background and Objective:**

This systematic review with meta-analysis investigated the influence of resistance training proximity-to-failure on muscle hypertrophy.

**Methods:**

Literature searches in the PubMed, SCOPUS and SPORTDiscus databases identified a total of 15 studies that measured muscle hypertrophy (in healthy adults of any age and resistance training experience) and compared resistance training performed to: (A) momentary muscular failure versus non-failure; (B) set failure (defined as anything other than momentary muscular failure) versus non-failure; or (C) different velocity loss thresholds.

**Results:**

There was a trivial advantage for resistance training performed to set failure versus non-failure for muscle hypertrophy in studies applying any definition of set failure [effect size=0.19 (95% confidence interval 0.00, 0.37), *p*=0.045], with no moderating effect of volume load (*p*=0.884) or relative load (*p*=0.525). Given the variability in set failure definitions applied across studies, sub-group analyses were conducted and found no advantage for either resistance training performed to momentary muscular failure versus non-failure for muscle hypertrophy [effect size=0.12 (95% confidence interval −0.13, 0.37), *p*=0.343], or for resistance training performed to high (>25%) versus moderate (20–25%) velocity loss thresholds [effect size=0.08 (95% confidence interval −0.16, 0.32), *p*=0.529].

**Conclusion:**

Overall, our main findings suggest that (i) there is no evidence to support that resistance training performed to momentary muscular failure is superior to non-failure resistance training for muscle hypertrophy and (ii) higher velocity loss thresholds, and theoretically closer proximities-to-failure do not always elicit greater muscle hypertrophy. As such, these results provide evidence for a potential non-linear relationship between proximity-to-failure and muscle hypertrophy.

**Supplementary Information:**

The online version contains supplementary material available at 10.1007/s40279-022-01784-y.

## Key Points

**Table:** 

This systematic review with meta-analysis grouped studies investigating the influence of resistance training proximity-to-failure on muscle hypertrophy into three broad themes based on the definition of set failure used (and therefore the specific research question being examined), to improve the validity of the meta-analyses.
Based on the limited available literature, our main findings show (i) no evidence to support that resistance training performed to momentary muscular failure is superior to non-failure resistance training, (ii) that higher velocity loss thresholds, and thus, theoretically closer proximities-to-failure, elicit greater muscle hypertrophy in a non-linear manner and (iii) no moderating effect of either volume load or relative load on muscle hypertrophy when resistance training was performed using any definition of set failure versus non-failure.
These findings provide evidence for a potential non-linear relationship between proximity-to-failure and muscle hypertrophy, but current set termination methods used during non-failure resistance training conditions limit insight into the actual proximity-to-failure achieved and pose a challenge for deriving practical recommendations for manipulating resistance training proximity-to-failure to achieve desired outcomes.

## Introduction

Resistance training (RT) promotes skeletal muscle hypertrophy, a physiological adaptation involving the structural remodelling of muscle tissue that leads to an increase in muscle fibre, and ultimately, whole-muscle cross-sectional area [1]. Although multiple RT variables (e.g. volume, load, frequency, lifting velocity) influence muscle hypertrophy, ‘proximity-to-failure’ specifically influences the exposure of muscle fibres to *mechanical tension*, the key stimulus for muscle hypertrophy [2]. Proximity-to-failure is defined as the number of repetitions remaining in a set prior to *momentary muscular failure* (i.e. when an individual cannot complete the concentric portion of a given repetition with a full range-of-motion without deviation from the prescribed form of the exercise) [3]. As proximity-to-failure nears within a given set, more repetitions are completed [thus increasing volume load (sets × repetitions × load)] and muscle fibre activation progressively increases [4, 5], ultimately exposing type II muscle fibres (capable of greater hypertrophy than type I muscle fibres [6]) to greater mechanical tension. However, whether the increased mechanical tension and volume load within a given set are worth the additional neuromuscular fatigue from reaching momentary muscular failure over multiple sets is contentious, as cumulative neuromuscular fatigue could impede the total volume load completed within an entire session or from session-to-session, and therefore decrease the total exposure to mechanical tension over time [3]. Nonetheless, inconsistencies in the literature limit understanding of the influence of RT proximity-to-failure on muscle hypertrophy and pose a challenge for deriving practical recommendations for manipulating proximity-to-failure during RT to achieve desired outcomes.

To our knowledge, three meta-analyses [7–9] investigated the influence of RT proximity-to-failure on muscle hypertrophy by comparing either RT performed to *set failure* (i.e. umbrella term describing the set termination criteria for the definition of ‘failure’ applied in a given study) versus non-failure [7, 8] or RT performed to different velocity loss thresholds that indirectly influence proximity-to-failure [9]. Results showed that RT performed to set failure does not elicit superior muscle hypertrophy compared with non-failure RT when volume load is equated [7, 8]. Further, RT performed to a higher velocity loss (> 25%) was found to be superior to a lower velocity loss (≤ 25%) for muscle hypertrophy [9]. Although trivial differences in muscle hypertrophy were found between 20–25% and > 25% velocity loss conditions (across a small number of studies that were sub-analysed) [9], collectively, these data suggest that the relationship between proximity-to-failure and muscle hypertrophy is likely non-linear [10] or that it is moderated by other RT variables such as volume load [8]. One of the major limitations of these data, however, is that no consensus definition for ‘failure’ exists in the literature. As such, these meta-analyses compare studies applying various definitions of set failure that alter the RT stimulus achieved. These differences in the RT stimulus achieved could potentially confound the conclusions drawn as the true proximity-to-failure compared between set failure conditions across studies is likely inconsistent.

To summarise the available evidence regarding the influence of RT proximity-to-failure on muscle hypertrophy while addressing critical research limitations, we identified three broad themes of studies in our recent scoping review [3], based on the definition of set failure applied and the research question asked (Table 1). We tentatively concluded that RT to set failure is likely not superior to non-failure RT for promoting muscle hypertrophy [3], but it is uncertain if meta-analysing these data within the themes we identified would alter this conclusion. Therefore, because of the methodological limitations identified in the current literature, the influence of proximity-to-failure on muscle hypertrophy is unclear and requires further investigation.

**Table 1:** ‘Themes’ of studies investigating proximity-to-failure in resistance training

Theme	Criteria
A	Studies comparing a group(s) performing resistance training to momentary muscular failure to a non-failure group(s) [13, 17–20]
B	Studies comparing a group(s) performing resistance training to set failure (defined as anything other than the definition of momentary muscular failure) to a non-failure group(s) [11, 12, 21, 22]
C	Studies theoretically comparing different proximities-to-failure (i.e. applying different velocity loss thresholds that modulate set termination and albeit indirectly, influence proximity-to-failure), with no inclusion of a group performing resistance training to momentary muscular failure per se [14–16, 23–25]

Description of specific criteria used to allocate studies to each theme, based on the definition of set failure applied and the research questions asked

### Objectives

Since the publication of previous meta-analyses [7–9] on the influence of proximity-to-failure on muscle hypertrophy, six additional studies were published [11–16] on this topic. Thus, this systematic review with meta-analysis extends previous findings by including new evidence and grouping studies into broad themes exclusive to the definition of set failure applied and the research question asked (Table 1). Specifically, we estimated: (i) the overall effect of RT performed to set failure versus non-failure on muscle hypertrophy and the individual effect of (A) definitions applied to set failure (based on ‘theme’), (B) volume load and (C) relative load on muscle hypertrophy, (ii) whether the magnitude of velocity loss achieved during RT influences muscle hypertrophy, and (iii) the magnitude of muscle hypertrophy achieved when RT is performed to momentary muscular failure, to set failure, and to a high velocity loss.

## Methods

A systematic review with meta-analysis was performed in accordance with the Preferred Reporting Items for Systematic Reviews and Meta-Analyses (PRISMA) guidelines [26]. The original protocol was registered with the Open Science Framework on 27 April, 2022 (https://osf.io/rzn63/) but since was slightly adjusted to improve the suitability of the analysis with the data and research questions (we did not perform the pre-registered meta-regression analysis, described further in Sect. 2.7). Because of the heterogeneity of studies investigating the influence of proximity-to-failure, a scoping review was previously conducted as a means of summarising the available evidence [3]. The systematic search used in the scoping review was adopted for this systematic review with meta-analysis to provide a consistent and objective understanding of the data. To reduce bias during the process, two authors (MR and JF) were involved in each step of the study identification process (including the literature search and study screening/selection), subsequent data extraction and methodological quality assessment for this systematic review with meta-analysis, with any disagreement resolved by mutual discussion.

### Research Questions

The research questions were defined using the participants, interventions, comparisons, outcomes and study design (PICOS) framework, as follows. In apparently healthy adults of any age and training status:

1. What is the overall effect of RT performed to set failure versus non-failure on muscle hypertrophy? And what is the individual effect of the definitions applied to set failure (based on ‘theme’), volume load and relative load on muscle hypertrophy?
2. Does the magnitude of velocity loss achieved (and theoretically, the proximity-to- failure reached) during RT influence muscle hypertrophy?
3. What magnitudes of muscle hypertrophy are achieved when RT is performed to momentary muscular failure, to set failure and to a high velocity loss?

### Literature Search Strategy

As described in our previous scoping review [3], the literature search followed the PRISMA-ScR (Preferred Reporting Items for Systematic Reviews and Meta-Analyses for Scoping Reviews) guidelines [27]. Literature searches of the PubMed, SCOPUS and SPORTDiscus databases were conducted in September 2021, and the following PubMed search string was used and adapted for each individual database: (("resistance training" OR "resistance exercise" OR "strength training") AND ("failure" OR "muscular failure" OR "velocity loss") AND (("muscle hypertrophy" OR "muscle size" OR "muscle growth" OR "muscle mass" OR "muscle thickness" OR "cross-sectional area") OR ("fatigue" OR "neuromuscular fatigue" OR "peripheral fatigue" OR "muscle damage" OR "discomfort" OR “enjoyment” OR "affective" OR "affective response"))). Since the initial search, however, two recently published studies [15, 16] in 2022 have been manually added to this systematic review with meta-analysis and subjected to the same screening process as studies retrieved in the initial database search.

### Study Selection

Covidence (Veritas Health Innovations, Melbourne, VIC, Australia) was used to manage and conduct the systematic study selection process, including the removal of duplicates and the exclusion of ineligible studies at each stage of the screening process*.* The systematic literature search and study selection process were completed independently by two blinded (to reduce any bias during this process) authors (MR and JF) with any disagreement resolved by mutual discussion. Finally, the authors (MR and JF) reviewed the full text to determine eligibility for inclusion based on the inclusion criteria. If any papers were added through reference checking or manual searching, they were subjected to the same screening process as if they were found in the initial database search.

### Inclusion Criteria

Studies were included if: (1) participants were apparently healthy adults of any age and RT experience, (2) participants were randomised to experimental groups, (3) the experimental comparison involved a group performing RT to set failure (any definition of set failure) versus a non-failure group, or two groups terminating RT sets at different proximities-to-failure (e.g. set termination informed by velocity loss thresholds or subjective ratings of perceived exertion), (4) one of the following measures of muscle hypertrophy was included; (a) muscle thickness, (b) whole-limb or muscle cross-sectional area or volume, (c) muscle fibre cross-sectional area or (d) lean body/fat free mass via dual X-ray absorptiometry or bioelectrical impedance analysis. Only original research studies in peer-reviewed journals were included, and studies were excluded if they involved (i) advanced set strategies (e.g. rest-pause, cluster sets), (ii) extraneous training variables (e.g. aerobic exercise, blood flow restriction), (iii) outcome measures that were not relevant and (iv) data that were duplicated within another included study.

### Data Extraction

Data charting was carried out by two authors (MR and JF) to capture key information in a table format (Table 2). The following participant characteristics were extracted: (1) RT status (i.e. untrained or resistance trained), (2) age and (3) sex. The following study characteristics were also extracted: (1) first author, (2) sample size, (3) publication date and (4) intervention groups/protocol outlines and duration. Raw data (mean and standard deviation) from pre-intervention and post-intervention for muscle hypertrophy outcomes were also extracted from each individual study for generation of standardised mean differences, confidence intervals (CIs) and subsequent meta-analysis. If figures were used instead of numerical data, those data were extracted from the figures using Web Plot Digitizer, and if the mean and standard deviation data were not reported, we contacted the authors of the respective study directly to obtain the relevant data. Our previous scoping review [3] identified three broad study themes across the relevant literature, and as such, each included study was grouped into one of the themes based on the criteria outlined in Table 1.

**Table 2:** Summary of data extraction. Brief summary of all studies including in this systematic review with meta-analysis

Study	Theme	Sample size (*n*)	Sex	Age (y)	Intervention groups/duration (sessions/week)	Volume equated	Training status
Lacerda et al. 2020 [17]	A	10	M	23.7 ± 4.9	**Failure:** 3–4 sets × *n* reps (50–60% 1-RM) **Non-failure:** 3–4 sets × *n* reps (50–60% 1-RM) → 14 weeks (2–3/week)	Yes	UT
Lasevicius et al. 2019 [20]	A	25	M	24 ± 4.9	**Failure 1:** 3 sets × *n* reps (80% 1-RM) **Failure 2:** 3 sets × *n* reps (30% 1-RM) **Non-failure 1:** ~ 5 sets × *n* reps (80% 1-RM) **Non-failure 2:** ~ 5 sets × *n* reps (30% 1-RM) → 8 weeks (2/week)	Yes	UT
Martorelli et al. 2017 [18]	A	89	F	21.9 ± 3.3	**Failure:** 3 sets × *n* reps (70% 1-RM) **Non-failure 1:** 4 sets × 7 reps (70% 1-RM) **Non-failure 2:** 3 sets × 7 reps (70% 1-RM) → 10 weeks (2/week)	Yes No	UT
Nobrega et al. 2018 [19]	A	32	M	23 ± 3.6	**Failure 1:** 3 sets × *n* reps (80% 1-RM) **Failure 2:** 3 sets × *n* reps (30% 1-RM) **Non-failure 1:** 3 sets × *n* reps to VI (80% 1-RM) **Non-failure 2:** 3 sets × *n* reps to VI (30% 1-RM) → 12 weeks (2/week)	Yes	UT
Santanielo et al. 2020 [13]	A	14	M	23.1 ± 2.2	**Failure:** *n* sets × *n* reps (75% 1-RM) **Non-failure:** *n* sets × *n* reps to VI (75% 1-RM)	No	T
Bergamasco et al. 2020 [12]	B	41	M/F	65.5 ± 4.5	**Failure:** 3 sets × *n* reps (40% 1-RM) **Non-failure 1:** 3 sets × *n* reps to VI (40% 1-RM) **Non-failure 2:** 3 sets × 10 reps (40% 1-RM) → 12 weeks (2/week)	No	UT
Karsten et al. 2021 [21]	B	18	M	23.5 ± 4.5	**Failure:** 4 sets × 10-RM (75% 1-RM) **Non-failure:** 8 sets × 5 reps (75% 1-RM) → 6 weeks (2/week)	Yes	T
Sampson et al. 2016 [22]	B	28	M	23.8 ± 6.6	**Failure:** 4 sets × 6 reps (85% 1-RM) **Non-failure 1**: 4 sets × 4 reps (85% 1-RM) **Non-failure 2**: 4 sets × 4 reps (85% 1-RM) → 12 weeks (3/week)	No	UT
Terada et al. 2021 [11]	B	27	M	20.03 ± 0.8	**Failure:** 3 sets × *n* reps to VF (40% 1-RM) **Non-failure:** 3 sets × 20% VeL (40% 1-RM) → 8 weeks (2/week)	Yes	UT
Andersen et al. 2021 [14]	C	10	M/F	23.0 ± 4.3	**High VeL:** 2–3 sets × 30% VeL (75–80% 1-RM) **Low VeL:** 4–6 sets × 15% VeL (75–80% 1-RM) → 9 weeks (2/week)	Yes	T
Pareja-Blanco et al. 2017 [25]	C	24	M	22.7 ± 1.9	**High VeL:** 3 sets × 40% VeL (70–85% 1-RM) **Mod VeL:** 3 sets × 20% VeL (70–85% 1-RM) → 8 weeks (2/week)	No	T
Pareja-Blanco et al. 2020 [24]	C	64	M	24.1 ± 4.3	**High VeL:** 3 sets × 40% VeL (70–85% 1-RM) **Mod VeL:** 3 sets × 20% VeL (70–85% 1-RM) **Low VeL:** 3 sets × 10% VeL (70–85% 1-RM) **Low VeL:** 3 sets × 0% VeL (70–85% 1-RM) → 8 weeks (2/week)	No	T
Pareja-Blanco et al. 2020 [23]	C	64	M	24.1 ± 4.3	**High VeL:** 3 sets × 50% VeL (70–85% 1-RM) **Mod VeL:** 3 sets × 25% VeL (70–85% 1-RM) **Low VeL:** 3 sets × 15% VeL (70–85% 1-RM) **Low VeL:** 3 sets × 0% VeL (70–85% 1-RM) → 8 weeks (2/week)	No	T
Rissanen et al. 2022 [15]	C	45	M/F	25.95 ± 3.85	**High VeL:** 2–5 sets × 40% VeL (65–75% 1-RM) **Mod VeL:** 2–5 sets × 20% VeL (65–75% 1-RM) → 8 weeks (2/week)	No	T
Rodiles-Guerrero et al. 2022 [16]	C	50	M	23.3 ± 3.3	**High VeL:** 3 sets × 50% VeL (55–70% 1-RM) **Mod VeL:** 3 sets × 25% VeL (55–70% 1-RM) **Low VeL:** 3 sets × 15% VeL (55–70% 1-RM) **Low VeL:** 3 sets × 0% VeL (55–70% 1-RM) → 8 weeks (2/week)	No	T

Studies were grouped into broad themes that involved resistance training performed to Theme A momentary muscular failure versus non-failure, Theme B set failure (defined as anything other than momentary muscular failure) versus non-failure or Theme C different velocity loss thresholds

*F* female, *M* male, *reps* repetitions, *RM* repetition maximum, *T* trained, *UT* untrained, *VeL* velocity loss, *VF* volitional failure, *VI* volitional interruption, *y* years

### Methodological Quality Assessment

Evaluation of methodological study quality (including risk of bias) was conducted by two authors (MR and JF) using the tool for the assessment of study quality and reporting in exercise (TESTEX) scale [28] shown in Table S1 of the Electronic Supplementary Material (ESM). The TESTEX scale is an exercise science-specific scale used to assess the quality and reporting of exercise training trials. The scale contains 12 criteria that can either be scored a ‘one’ or not scored at all; 1, eligibility; 2, randomisation; 3, allocation concealment; 4, groups similar at baseline; 5, assessor blinding; 6, outcome measures assessed in 85% of patients (3 possible points); 7, intention-to-treat; 8, between-group statistical comparisons (2 possible points); 9, point estimates of all measures included; 10, activity monitoring in control groups; 11, relative exercise intensity remained constant; 12, exercise parameters recorded. The best possible total score is 15 points.

### Statistical Analysis

All statistical analyses were conducted with the ‘metafor’ [29] package in R (version 4.0.2; R Core Team, https://www.r-project.org/) and all of the code utilised is openly available. Standardised effect sizes (ESs) and standard errors were calculated using the ‘escalc’ function in ‘metafor’. The magnitude of standardised ESs was interpreted with reference to Cohen’s *d* (1988) thresholds: trivial (< 0.2), small (0.2 to < 0.5), moderate (0.5 to < 0.8) and large (> 0.8). Point estimates and their 95% CIs were produced. Restricted maximal likelihood estimation was used in all models. Given that correlations between pre-test and post-test measures are rarely reported in original studies, a correlation coefficient of *r* = 0.75, which was replicated from Grgic et al. [7], was used to calculate the variance (or standard error) for all studies and sensitivity analyses were performed using correlation coefficients that ranged from *r* = 0.6 to *r* = 0.9 (Figs. S1–S4 of the ESM). Funnel plots were generated (Figs. S5–S6 of the ESM) and Egger’s test was applied to assess the risk of bias from small-study effects. The *I*^2^ heterogeneity statistic was also produced and reported to indicate the proportion of the observed variance (for all ESs generated) that is not due to sampling error [30]. To complement traditional null hypothesis significance testing, we also considered the practical implications of all results by qualitatively assessing the ES estimate and associated CI width.

A quantitative synthesis of studies in Theme A and B (combined), and Theme C, was performed using a multi-level mixed-effects meta-analysis, as there is a nested structure to the ESs that were calculated from the studies included (i.e. multiple ESs from various measures of muscle hypertrophy nested within groups and nested within studies). Standardised ESs were calculated such that a positive ES favours the set failure conditions (or high velocity loss conditions), whereas a negative ES favours non-failure conditions (or moderate velocity loss conditions). A multi-level model for studies in Theme A and B was produced including all standardised ESs to provide a general estimate of the effect and to answer review question one. Studies from Theme A and B were also categorised by: (i) theme (A or B), (ii) the difference in volume load between set failure and non-failure conditions (volume equated or not volume equated) and (iii) the relative load lifted [high load (> 50% 1-repetition maximum [RM]) or low load (≤ 50% 1-RM)], and sub-group analyses were employed to estimate an ES for the influence of these individual variables (i.e. theme, volume load, relative load) on the outcome measure and compare and contrast the estimates. Another multi-level model was produced for studies in Theme C comparing high velocity loss conditions (> 25%) versus moderate velocity loss conditions (20–25%), to provide a general estimate of the effect and help answer review question two. Three [16, 23, 24] out of the six studies [14–16, 23–25] in Theme C also involved groups performing RT to low velocity loss thresholds (< 20%); however, considering only six ESs could be retrieved (vs 11 ESs for both moderate and high velocity loss thresholds) and the low practical importance of performing RT with < 20% velocity loss, we excluded low velocity loss conditions from this comparative model and therefore did not perform the pre-registered meta-regression analysis (https://osf.io/rzn63/). However, an individual standardised ES was calculated for the low velocity loss conditions, along with all other RT conditions analysed [i.e. momentary muscular failure, set failure, non-failure, and moderate (20–25%) and high (> 25%) velocity loss thresholds] across all studies in each theme to provide a general estimate of the effect and help answer review questions two and three.

## Results

### Search Results and Systematic Review of Included Studies

**Fig. 1:**
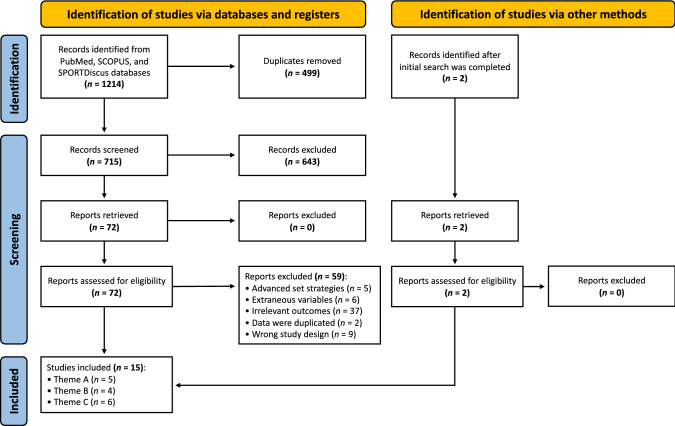
PRISMA (Preferred Reporting Items for Systematic Reviews and Meta-Analyses) flow chart. Summary of the systematic literature search and study selection process

The original literature search results were described previously [3], and an updated flowchart of the systematic literature search and study selection process is displayed in Fig. 1. For this systematic review with meta-analysis, two additional studies [15, 16] were found through manual checking and were subject to the same screening process as studies retrieved in the initial database search. Further, all studies retrieved from the original search that did not measure muscle hypertrophy outcomes were excluded from this systematic review with meta-analysis, leaving a total of 15 studies eligible for analysis. Subsequently, studies were grouped into one of the three themes identified based on the criteria outlined in Table 1 to improve the validity of study comparisons and interpretations within each theme. Results from Egger’s test found no publication bias (*p* < 0.05) for studies in Theme A and B, and studies in Theme C. For a summary of included studies, see Table 2.

A total of nine studies [11–13, 17–22] compared RT performed to set failure (including all definitions of set failure) versus non-failure and measured muscle hypertrophy in one or more of the following muscle groups: quadriceps (vastus lateralis, vastus medialis, rectus femoris), elbow flexor, triceps brachii, pectoralis major or anterior deltoid. Five [13, 17–20] out of the nine [11–13, 17–22] studies applied the definition of momentary muscular failure and were thus allocated to Theme A, and the remaining four studies [11, 12, 21, 22] applied various definitions of set failure other than momentary muscular failure and were thus allocated to Theme B. Importantly, five [11, 17, 19–21] out of the nine studies [11–13, 17–22] equated volume load between conditions, whereas three studies [12, 13, 22] did not equate volume load. The final study [18] involved two non-failure conditions, of which one was volume equated (compared to the set failure condition), while the other was not. Further, five [13, 17, 18, 21, 22] out of the nine studies [11–13, 17–22] used a high load (> 50% 1-RM), and two studies [11, 12] used a low load (≤ 50% 1-RM). The remaining two studies [19, 20] used both high and low loads allocated across two set failure and two non-failure conditions. Of the five studies in Theme A, four studies [13, 17, 19, 20] found no statistically significant differences between conditions in muscle hypertrophy from pre-intervention to post-intervention, while one study [18] did not perform a between-group statistical analysis. Similarly, three [11, 21, 22] of the four studies [11, 12, 21, 22] in Theme B found no statistically significant differences in muscle hypertrophy between conditions, and one study [12] found no statistically significant pre-intervention to post-intervention changes in muscle size for either condition. A total of seven studies [11, 12, 17–20, 22] from both Theme A and B involved untrained participants, whereas only two studies involved resistance-trained participants [13, 21].

Additionally, a total of six studies [14–16, 23–25] in resistance-trained participants compared high velocity loss conditions (> 25%) with moderate velocity loss conditions (20–25%) and measured muscle hypertrophy (Theme C) in one or more of the following muscle groups: quadriceps (vastus lateralis, vastus intermedius, vastus medialis, rectus femoris) or pectoralis major. Five [14, 15, 23–25] out of the six [14–16, 23–25] studies in Theme C observed increases in muscle hypertrophy when RT was performed to both high and moderate velocity loss; however, no statistically significant differences between conditions were found in each of the studies. The remaining study [16] only found increases in muscle hypertrophy for the high velocity loss condition. All studies [14–16, 23–25] in Theme C involved a high load and were conducted on resistance-trained participants.

### Methodological Quality

A detailed overview of the methodological quality of included studies using the TESTEX scale [16] can be found in Table S1 of the ESM. Study quality scores ranged from 7 to 12 (out of a possible 15), with mean and median scores of 9.9 and 10, respectively (Table S1 of the ESM). Although each study had some risk of bias, all studies lost two points because of (i) no allocation concealment and (ii) no activity monitoring, and only one study clearly stated if an ‘intention-to-treat’ analysis was performed on outcomes of interest. Overall, a total of 11 out of 15 studies scored highly (> 10) on the TESTEX scale and visual inspection of methodological quality results revealed no impact of study quality on the ES estimates generated.

### Meta-analysis Results

#### What is the Overall Effect of Resistance Training Performed to Set Failure (Irrespective of the Definition Applied) Versus Non-Failure on Muscle Hypertrophy?

**Fig. 2:**
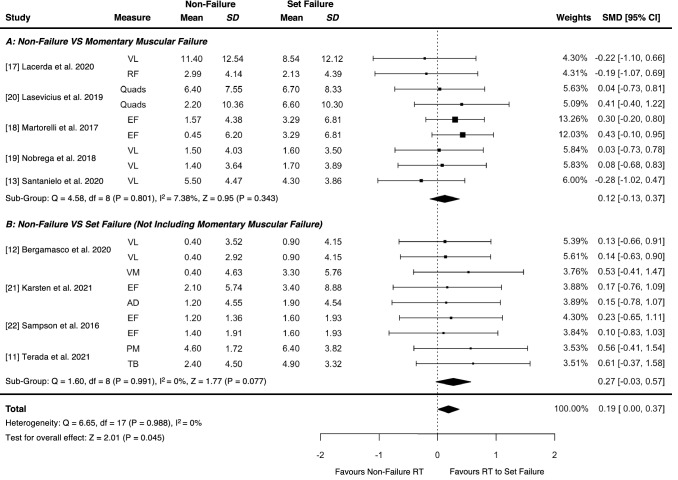
Influence of resistance training (RT) performed to set failure versus non-failure on muscle hypertrophy with subgroup analyses based on study ‘theme’ (A or B). Studies presented were grouped into broad themes that involved RT performed to either momentary muscular failure versus non-failure (Theme A), or set failure (defined as anything other than momentary muscular failure) versus non-failure (Theme B). Point estimates and error bars signify the standardised mean difference between set failure and non-failure conditions and 95% confidence interval (CI) values, respectively. *AD* anterior deltoid, *EF* elbow flexors, *PM* pectoralis major, *Quads* quadriceps, *RF* rectus femoris, *SD* standard deviation, *SMD* standardized mean difference, *TB* triceps brachii, *VL* vastus lateralis, *VM* vastus medialis

Meta-analytic outcomes for the overall effect of RT performed to set failure (irrespective of the definition applied) versus non-failure on muscle hypertrophy from all studies in Theme A and B are shown in Fig. 2. There was a statistically significant advantage for RT performed to set failure versus non-failure on muscle hypertrophy, which was trivial in magnitude [ES = 0.19 (95% CI 0.00, 0.37), *p* = 0.045] with a very low heterogeneity (*Q* = 6.65, *p* = 0.988, *I*^2^ = 0%).

##### Influence of Volume Load, Relative Load and the Definition of Set Failure on Muscle Hypertrophy Following Resistance Training Performed to Set Failure Versus Non-Failure

Outcomes for sub-group analyses of studies categorised into either Theme A or Theme B are shown in Fig. 2. Sub-group analysis of studies applying the definition of momentary muscular failure (Theme A) found no statistically significant difference between RT performed to momentary muscular failure and non-failure on muscle hypertrophy, with a trivial standardised effect [ES = 0.12 (95% CI − 0.13, 0.37), *p* = 0.343] involving a low heterogeneity (*Q* = 4.58, *p* = 0.801, *I*^2^ = 7.38%). Similar results were found when analysing studies that applied definitions of set failure other than momentary muscular failure (Theme B), with no statistically significant difference between RT performed to set failure (not including momentary muscular failure) and non-failure on muscle hypertrophy, with a trivial standardised effect [ES = 0.27 (95% CI − 0.03, 0.57), *p* = 0.077] involving a very low heterogeneity (*Q* = 1.60, *p* = 0.991, *I*^2^ = 0%). Individual ESs were calculated for subgroups categorised by volume load standardisation (equated vs not equated) and relative load lifted (higher load vs lower load); these pooled ESs are presented in Table 3. Moderator analyses revealed that neither volume load standardisation (*p* = 0.884) nor relative load lifted (*p* = 0.525) had statistically significant impacts on the overall ES for muscle hypertrophy.

**Table 3:** Influence of volume load and relative load on muscle hypertrophy outcomes in response to resistance training performed to set failure versus non-failure

Sub-group analysis	Classification	ES (95% CI)	*p*-value
Volume load	Volume equated	0.20 (− 0.03, 0.43)	0.09
	Not volume equated	0.17 (− 0.13, 0.47)	0.27
Relative load	Higher load (> 50% 1-RM)	0.15 (− 0.07, 0.37)	0.18
	Lower load (≤ 50% 1-RM)	0.28 (− 0.06, 0.62)	0.11

Data shown are presented as a standardised ES estimate (signifying the standardised mean difference between set failure and non-failure conditions) with 95% CI and associated *p*-value. Positive ES values favour resistance training performed to set failure versus non-failure

*CI* confidence interval, *ES* effect size, *RM* repetition maximum

#### Does the Magnitude of Velocity Loss Achieved (and Theoretically, the Proximity-to-Failure Reached) During Resistance Training Influence Muscle Hypertrophy?

**Fig. 3:**
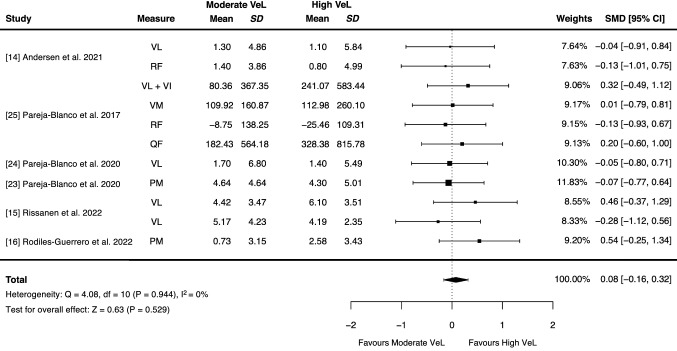
Influence of resistance training performed to high (> 25%) and moderate (20–25%) velocity loss on muscle hypertrophy based on studies in Theme C. Studies presented were grouped into Theme C that involved resistance training performed to different velocity loss thresholds. *Point estimates* and *error bars* signify the standardised mean difference (SMD) between high and moderate velocity loss conditions and 95% confidence interval (CI) values, respectively. *PM* pectoralis major, *QF* quadriceps femoris, *RF* rectus femoris, *SD* standard deviation, *VeL* velocity loss, *VI* vastus intermedius, *VL* vastus lateralis, *VM* vastus medialis

**Fig. 4:**
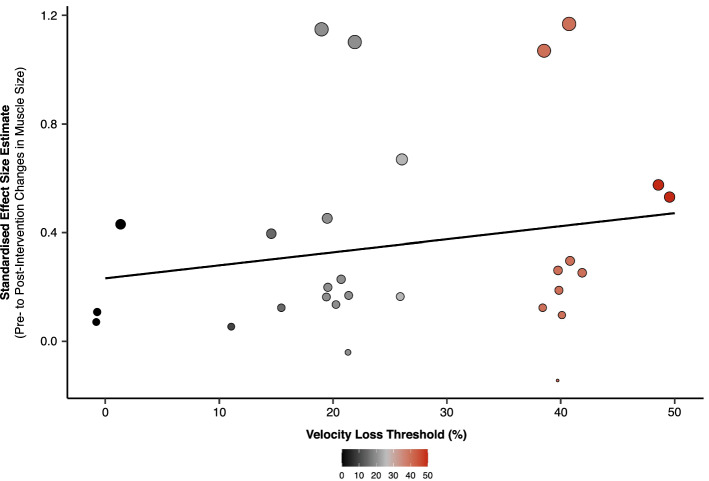
Individual standardised effect sizes (pre-intervention to post-intervention changes in muscle size) for all velocity loss conditions [low (< 20%), moderate (20–25%), high (> 25%)] in each study from Theme C. Data presented were extracted from studies grouped into Theme C that involved resistance training performed to different velocity loss thresholds. The *size* of the *dot point* is based on a standardised effect size and a horizontal ‘jitter’ was applied to limit the overlap of dot points (as such, the dot point position on the *x-axis* is not a true representation of the velocity loss achieved and is rather limited to 0, 10, 15, 20, 25, 40 and 50% velocity losses). Positive effect size values indicate increases in muscle size from pre-intervention to post-intervention for each velocity loss condition

Meta-analytic outcomes for the influence of high (> 25%) and moderate (20–25%) velocity loss thresholds on muscle hypertrophy are shown in Fig. 3. Results of the multi-level meta-analysis model indicated no statistically significant difference between high velocity loss and moderate velocity loss conditions on muscle hypertrophy, revealing a trivial standardised effect [ES = 0.08 (95% CI − 0.16, 0.32)*, p* = 0.529] with a very low heterogeneity (*Q* = 4.08, *p* = 0.944, *I*^2^ = 0%). Individual standardised ESs for velocity loss conditions in each study from Theme C are displayed in Fig. 4. Velocity loss conditions were also categorised as low (< 20%), moderate (20–25%) or high (> 25%), and the mean values and CIs for each velocity loss condition are also shown in Table 4.

**Table 4:** Individual standardised ESs for resistance training conditions across all studies in each ‘theme’ (A, B and C)

Theme	Condition	ES (95% CI)	*p*-value
A	Momentary muscular failure	0.41 (0.27, 0.55)	< 0.001
	Non-failure	0.37 (0.15, 0.58)	0.001
B	Set failure	0.46 (0.12, 0.80)	0.077
	Non-failure	0.32 (0.05, 0.60)	0.023
C	Low velocity loss (< 20%)	0.20 (− 0.02, 0.41)	0.072
	Moderate velocity loss (20–25%)	0.39 (0.09, 0.70)	0.010
	High velocity loss (> 25%)	0.42 (0.12, 0.71)	0.005

Data shown are presented as a standardised ES estimate (signifying the standardised mean difference for pre-intervention to post-intervention changes in muscle size for each resistance training condition) with 95% CI and associated *p*-value

*ES* effect size, *CI* confidence interval

#### What Magnitudes of Muscle Hypertrophy Are Achieved When Resistance Training is Performed to Momentary Muscular Failure (Theme A), to Set Failure (Theme B) and to a High Velocity Loss (Theme C; 40 or 50% Velocity Loss)?

Individual standardised effect sizes for pre-intervention to post-intervention changes in muscle size for each RT condition (i.e. momentary muscular failure, set failure, non-failure, and low, moderate and high velocity loss thresholds) across all studies in each ‘theme’ (A, B and C) are shown in Table 4.

### Sensitivity-Analysis Results

Sensitivity analyses were performed for all multi-level meta-analysis models, with correlation coefficients that ranged from *r* = 0.6 to *r* = 0.9 (per hundredth decimal), to assess whether the selected correlation coefficient (*r* = 0.75) influenced the meta-analytic outcomes (Figs. S1–S4 of the ESM). For the meta-analysis estimating the overall effect of RT performed to set failure versus non-failure on muscle hypertrophy (Sect. 3.3.1), ESs between 0.15 and 0.25 and *p*-values between 0.016 and 0.104 were observed. Although our meta-analysis found a statistically significant effect (*p* = 0.045) of RT performed to set failure versus non-failure on muscle hypertrophy, this result should be interpreted with caution. In accordance with the previous literature [7], this analysis was conducted with an a priori assumption that the correlation coefficient between pre-test and post-test measures was *r* = 0.75; while this is a defensible assumption, sensitivity analyses revealed outcomes that were not statistically significant with correlation coefficients below *r* = 0.73 (Fig. S1 of the ESM). Conversely, the meta-analysis that compared studies in Theme C to determine whether the magnitude of velocity loss influenced muscle hypertrophy (Sect. 3.3.2) observed ES and *p*-value ranges of 0.06–0.11 and 0.356–0.612, respectively. Our meta-analysis found no statistically significant difference between high velocity loss and moderate velocity loss conditions on muscle hypertrophy (*p* = 0.529) and considering that no statistically significant *p*-values were observed (*p* < 0.05) across the range of correlation coefficients analysed (Fig. S3 of the ESM), the results of this meta-analysis may be interpreted with increased confidence.

## Discussion

### Influence of Resistance Training Performed to Set Failure (Including Momentary Muscular Failure and Other Definitions) Versus Non-Failure on Muscle Hypertrophy

A key barrier to further understanding the influence of proximity-to-failure on muscle hypertrophy is that no consensus definition for set failure exists in the literature. Previous meta-analyses [7, 8] compared studies that involved various definitions of set failure, and no statistically significant differences between RT performed to ‘failure’ versus non-failure on muscle hypertrophy were found. However, because of the heterogeneity in proximities-to-failure achieved, these results may not provide an accurate insight into the true effect of reaching momentary muscular failure during RT, which is the most objective means of defining set failure [3].

Similar to previous meta-analyses [7, 8], we first aimed to estimate the overall effect of RT performed to set failure (irrespective of the definition applied) versus non-failure on muscle hypertrophy. We also investigated whether the definition of set failure applied influenced the results. In our analysis of studies that applied any definition of set failure (Theme A and B), we found a trivial advantage for RT performed to set failure versus non-failure on muscle hypertrophy [Fig. 2; ES = 0.19 (95% CI 0.00, 0.37), *p* = 0.045]. These findings contrasted with previous meta-analytic results [7, 8]; however, because of the aforementioned limitations of this approach and the results of our sensitivity analysis (Sect. 3.4), the validity of these results is uncertain. Of greater importance is our sub-group analysis of studies that applied the definition of momentary muscular failure (Theme A) and found no evidence to support that RT performed to momentary muscular failure is superior to non-failure RT for muscle hypertrophy [Fig. 2; ES = 0.12 (95% CI − 0.13, 0.37), *p* = 0.343]. Indeed, the definition of momentary muscular failure involves involuntary set termination and is the only approach to standardise the RT stimulus both within and between studies when RT is performed to ‘failure’. Thus, applying the definition of momentary muscular failure likely improves the validity of outcomes as demonstrated by a narrower CI width (i.e. lower uncertainty) [Table 4; ES = 0.41 (95% CI 0.27, 0.55)] compared with when RT is performed to set failure (definitions other than momentary muscular failure) and the true proximity-to-failure achieved likely varies [Table 4; ES = 0.46 (95% CI 0.12, 0.80)]. Our sub-group analysis of studies that did not apply the definition of momentary muscular failure (Theme B) also demonstrated no statistically significant difference between conditions [Fig. 2; ES = 0.27 (95% CI − 0.03, 0.57), *p* = 0.077] and it is likely that these studies simply compared different proximities-to-failure, therefore preventing inferences about the specific effect of reaching momentary muscular failure on muscle hypertrophy. Although differences in CI width between our sub-group analyses (Theme A vs Theme B) may be due to the definition of set failure applied, considerable variability and ambiguity in the proximity-to-failure achieved in non-failure RT conditions also exists within the literature, which likely also contributes to differences in the ES estimates observed for pre-intervention to post-intervention changes in muscle size and their associated CIs. To reiterate, despite finding a trivial advantage for RT performed to set failure versus non-failure on muscle hypertrophy when meta-analysing studies that applied any definition of set failure, our sub-group analyses that evaluated studies based on the definition of set failure applied found (i) no advantage of performing RT to momentary muscular failure versus non-failure on muscle hypertrophy and (ii) that closer proximities-to-failure do not always elicit greater muscle hypertrophy. Overall, this analysis demonstrated that skeletal muscle can be effectively stimulated to hypertrophy prior to reaching momentary muscular failure during RT, but because of methodological limitations, it is difficult to discern the proximity-to-failure that would theoretically maximise muscle hypertrophy.

#### Effect of Volume Load on the Influence of Proximity-to-Failure on Muscle Hypertrophy

We also generated a sub-group analysis on all studies (irrespective of the definition of set failure applied) to assess whether volume load moderated the influence of proximity-to-failure on muscle hypertrophy. We found similar ES estimates (and CI width) for muscle hypertrophy between set failure (irrespective of the definition applied) and non-failure conditions in studies that equated volume load [Table 3; ES = 0.20 (95% CI − 0.03, 0.43)], and those that did not equate volume load [Table 3; ES = 0.17 (95% CI − 0.13, 0.47)]. These findings support the idea that equating volume load between conditions may be unnecessary when evaluating the effect of proximity-to-failure on muscle hypertrophy. Rather, it remains possible that set volume (i.e. the number of sets performed to, or close to, momentary muscular failure per muscle group per week [31]), which was equated between conditions in seven [11–13, 17–19, 22] out of the nine [11–13, 17–22] studies, has a more potent effect on muscle hypertrophy than volume load [31]. Although our analysis found no moderating effect of volume load on the overall ES for muscle hypertrophy (*p* = 0.884), the effect of volume load as a moderator variable is limited by the set volume prescribed in research interventions, which may be lower than set volumes commonly achieved in practice [32]. Considering the similarities in set volume completed across studies included in our meta-analysis, it is also unlikely that set volume had a moderating effect on the overall ES for muscle hypertrophy. As such, future research investigating the effect of proximity-to-failure on muscle hypertrophy should thus (i) focus on equating set volume between conditions, (ii) investigate whether the number of sets performed for a given muscle group/exercises moderates the influence of proximity-to-failure on muscle hypertrophy and (iii) employ set volumes that reflect current scientific guidelines for best practice [33] to improve the practical recommendations derived.

#### Effect of Relative Load on the Influence of Proximity-to-Failure on Muscle Hypertrophy

Our sub-group analysis on studies that employed any definition of set failure also assessed whether the relative load lifted moderated the influence of proximity-to-failure on muscle hypertrophy. We found a larger ES estimate for muscle hypertrophy favouring set failure (irrespective of the definition applied) compared with non-failure conditions when lower loads were employed [≤ 50% 1-RM; ES = 0.28 (95% CI − 0.06, 0.62)] versus higher loads [> 50% 1-RM; ES = 0.15 (95% CI − 0.07, 0.37)]. Differences in CI width between loading conditions was likely owing to the variability in proximity-to-failure achieved amongst both set failure and non-failure conditions; particularly during lower load RT, as individuals are more likely to underestimate their proximity-to-failure when performing RT with lower loads versus higher loads [34], potentially because of the high levels of perceived discomfort that often accompany lower load RT [35]. Nonetheless, it is hypothesised that RT should be performed with a closer proximity-to-failure when lower loads are lifted versus higher loads. This strategy would theoretically maximise muscle fibre activation and subsequent muscle hypertrophy [36], and although the ES differences may provide support for this hypothesis, more research comparing lower load and higher load RT is required to elucidate the influence of relative load on muscle hypertrophy when RT is performed to different proximities-to-failure. Although we found no moderating effect of relative load on the overall ES for muscle hypertrophy (*p* = 0.525), future research should continue exploring the interaction of RT variables (e.g. set volume, relative load, exercise selection) with proximity-to-failure to foster insights that may improve RT prescription for muscle hypertrophy.

### Influence of Different Velocity Loss Thresholds on Muscle Hypertrophy

A recent meta-analysis investigated the effect of different velocity loss thresholds on muscle hypertrophy and found that velocity losses of > 25% (40% or 50% in all the analysed studies) were superior to velocity losses of ≤ 25% for muscle hypertrophy [9]; however, sub-analyses indicated that this result was largely driven by comparisons of higher velocity losses (40% and 50%) with those ≤ 20% as opposed to those between 20 and 25%. Considering the small number of studies employing velocity loss thresholds of < 20%, which likely confounded the validity of these sub-analyses, we therefore decided to define three velocity loss thresholds (low = < 20%, moderate = 20–25%, high = > 25%) and generated individual ESs for pre-intervention to post-intervention changes in muscle size for each velocity loss condition.

**Fig. 5:**
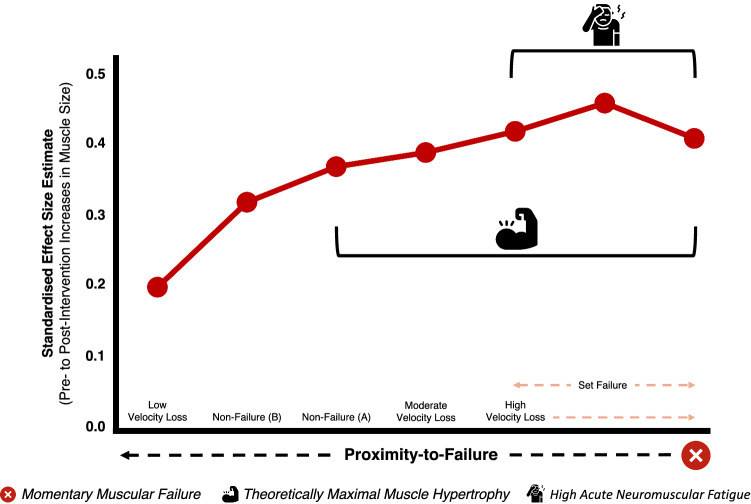
Conceptual non-linear relationship between proximity-to-failure and muscle hypertrophy. Our results suggest that closer proximities-to-failure are associated with muscle hypertrophy in a non-linear manner. Although the order of resistance training conditions displayed allows for visual inspection of a potential non-linear relationship between proximity-to-failure and muscle hypertrophy, the true proximities-to-failure achieved in each of these resistance training conditions are unclear and likely vary. The *far-right dot point* represents the ‘momentary muscular failure’ condition. It is also likely that participants in the ‘set failure’ and ‘high velocity loss’ conditions reached momentary muscular failure at times. Data shown are effect size estimates for pre-intervention to post-intervention increases in muscle size for each resistance training condition

Similar to the results of previous research [9], we found that higher velocity losses (20–50%), and theoretically, closer proximities-to-failure, were associated with greater muscle hypertrophy in a *non-linear* manner (Fig. 5). Smaller ES estimates for pre-intervention to post-intervention changes in muscle size were observed for the low velocity loss condition (ES = 0.20) versus the moderate (ES = 0.39) and high (ES = 0.42) velocity loss conditions, with meta-analytic results showing no advantage of performing RT to a high velocity loss (> 25%) versus a moderate velocity loss (20–25%) on muscle hypertrophy [Fig. 3 (ES = 0.08, 95% CI − 0.16 to 0.32*; p* = 0.529)]. While differences in velocity loss between conditions may provide indirect insights into the influence of proximity-to-failure on muscle hypertrophy, suggesting that closer proximities-to-failure during RT do not always elicit greater muscle hypertrophy, these findings should be interpreted with caution given the substantial variability in the proximity-to-failure achieved between individuals performing RT to the same velocity loss. For example, one study found that participants who performed the squat exercise until 40% velocity loss reached momentary muscular failure ~ 56% of the time [25], suggesting that the occurrence of momentary muscular failure likely varies between high velocity loss conditions across studies and contributes to the variability in muscle hypertrophy outcomes observed [highlighted by a relatively wide CI width for the high velocity loss threshold (95% CI 0.05, 0.76)].

Importantly, the results of our meta-analysis were found despite a greater volume load being accumulated when RT was performed to a high versus moderate velocity loss (e.g. the 40% velocity loss condition in one study performed over 100 repetitions more than the 20% velocity loss condition [9]), and although it has been claimed that differences in muscle hypertrophy between velocity loss conditions are due to differences in volume load [9], we propose that if velocity loss conditions of > 20% are compared (with set volume and relative load equated between conditions), differences in volume load have little to no additional impact on muscle hypertrophy in resistance-trained populations. As such, factors other than volume load (e.g. neuromuscular fatigue) may moderate the influence of proximity-to-failure on muscle hypertrophy when RT is performed to different velocity losses, or proximities-to-failure. Despite the limitations, relative differences in proximity-to-failure across different velocity loss thresholds remain and our findings provide evidence for a potential *non-linear relationship* between proximity-to-failure and muscle hypertrophy; however, future research that more accurately quantifies proximity-to-failure is required to better understand the relationship between proximity-to-failure and muscle hypertrophy.

### Practical Application of Key Findings

Our findings suggest that during RT, achieving a sufficient proximity-to-failure coupled with an adequate set volume for a given muscle group (approximately 12–20 sets performed per week on average [33]) are key determinants of muscle hypertrophy, rather than any specific benefit of performing RT to momentary muscular failure per se. Potentially contributing to the apparent non-linear relationship between proximity-to-failure and muscle hypertrophy may be the acute neuromuscular fatigue that rises as proximity-to-failure nears [3] and its implications on subsequent exposure to mechanical tension (Fig. 5). For example, in a given set, type II muscle fibres are likely exposed to higher levels of mechanical tension as proximity-to-failure nears, but high levels of acute neuromuscular fatigue may impair neural drive (via ‘central’ mechanisms [37, 38]) and/or excitation–contraction coupling (via ‘peripheral’ mechanisms [39, 40]), ultimately suppressing force production by type II muscle fibres and their exposure to mechanical tension over multiple sets (potentially observed as a decrease in the total repetitions performed with a given load from set-to-set, particularly when momentary muscular failure is reached [41]). This potential impairment of mechanical tension and subsequent muscle hypertrophy when high levels of acute neuromuscular fatigue are incurred may explain why RT performed to momentary muscular failure produces similar ES estimates (ES = 0.41) for pre-intervention to post-intervention changes in muscle size compared to set failure [irrespective of the definition applied] (ES = 0.46) and moderate (ES = 0.39) to high (ES = 0.42) velocity loss conditions.

Overall, RT prescription should not be treated dichotomously in practice and sets may be performed to both momentary muscular failure and non-failure in a given session of RT. For example, we suggest that a majority of RT sets are terminated with a close proximity-to-failure to limit the cumulative acute neuromuscular fatigue incurred and to maintain a high level of exposure to mechanical tension over multiple sets, with the decision to reach momentary muscular failure primarily based on safety and biased toward (i) exercises with low complexity and low associated fatigue (e.g. single-joint vs multi-joint exercises, exercises performed using machines versus free weights, exercises involving lower cardiovascular demands), (ii) the last set of a given exercise or muscle group, (iii) muscle groups involving a low set volume (< 5 sets per session) or weekly RT frequency (1–2 × per week), (iv) resistance-trained versus untrained individuals and (v) lower loads versus higher loads.

### Limitations

A total of 11 out of 15 studies scored highly (> 10) on the TESTEX scale and visual inspection of methodological quality results revealed no impact of study quality on the ES estimates generated. However, four studies did not state the percentage of participants who completed the study (i.e. did not withdraw), and five studies did not state the number of exercise sessions completed by participants who did not withdraw from study. The procedure used to randomise participants into intervention groups was also not described in eight studies, and no studies stated whether group allocation was concealed. Although it is unlikely that these limitations had a confounding influence on the outcomes of this review, future research should ensure that this information is clearly presented. Considering the correlation coefficients (*r* value) between pre-test and post-test measures are rarely reported in research studies, we assumed *r* = 0.75 to conduct our meta-analyses. Although this *r* value was replicated from a previous meta-analysis related to this topic [7], a sensitivity analysis suggests the results of our meta-analysis comparing set failure (irrespective of the definition applied) versus non-failure RT on muscle hypertrophy should be interpreted with caution, as outcomes of *p* > 0.05 were observed with correlation coefficients below *r* = 0.73. Furthermore, considering the relatively small body of available literature on the influence of RT proximity-to-failure on muscle hypertrophy, our meta-analytic results are likely confounded by statistical power limitations, particularly in our sub-group analyses. As such, although we found no supporting evidence that RT performed to momentary muscular failure is superior to non-failure RT for muscle hypertrophy, considering the low number of studies analysed, it is unclear if analysing a larger number of studies (and generating a greater statistical power) would alter this conclusion.

The results of our analyses may also be influenced by the current set termination methods used during set failure (not including momentary muscular failure) and non-failure RT conditions, which limit insight into the true proximity-to-failure achieved. For example, the proximity-to-failure achieved in these conditions likely varied within and between studies, and particularly when velocity loss thresholds were used to control set termination, as highlighted by the relatively wide CI width for our ES estimates (Table 4).

Overall, to improve the validity and practical applicability of results of future research investigating the influence of proximity-to-failure on muscle hypertrophy, researchers should (i) embrace thorough data reporting and dedication to open science so that future meta-analyses may start to use actual observed correlation coefficients (between pre-test and post-test measures), instead of estimating or assuming the *r* value, (ii) not treat the prescription of RT dichotomously (i.e. set failure or non-failure) and (ii) employ methods to control and report the proximity-to-failure reached during RT interventions.

## Conclusions

Our main findings show that: (i) RT performed to set failure is advantageous versus non-failure RT for muscle hypertrophy (trivial effect) when studies applying any definition of set failure are analysed; however, our sub-group analyses found no evidence to support that RT performed to momentary muscular failure [or to set failure (irrespective of the definition applied)] is superior to non-failure RT for muscle hypertrophy and (ii) higher velocity loss thresholds, and thus, theoretically closer proximities-to-failure, elicit greater muscle hypertrophy but in a non-linear manner. Although other RT variables may moderate the influence of proximity-to-failure on muscle hypertrophy, our findings revealed no effect of either volume load or relative load on muscle hypertrophy when RT was performed to set failure (using any definition) versus non-failure; however, larger ES estimates favouring RT to set failure were found for lower load versus higher load RT, providing some support for the idea that RT needs to be performed to closer proximities-to-failure when lower loads are lifted versus higher loads. Overall, these findings provide evidence for a potential non-linear relationship between proximity-to-failure and muscle hypertrophy. However, current methods used to control set termination during non-failure RT limit insight into the actual proximity-to-failure achieved, and as a result, the proximity-to-failure that would theoretically maximise muscle hypertrophy is unclear and requires further investigation.

### Supplementary Information

Below is the link to the electronic supplementary material.

Supplementary file1 (PDF 305 KB)

## Declarations

### Availability of data and material

All data are openly available on the Open Science Framework: https://osf.io/rzn63/.

### Code availability

The code utilised is openly available on the Open Science Framework: https://osf.io/rzn63/.
