# Supplementary material for: Influence of Resistance Training Proximity-to-Failure on Skeletal Muscle Hypertrophy: A Systematic Review with Meta-analysis
Source: Sports Med. 2022 Nov 5;53(3):649–65. doi: 10.1007/s40279-022-01784-y (PMC9935748; doi:10.1007/s40279-022-01784-y)
Supplement: Supplementary file 1 — Supplementary file1 (PDF 305 KB) [file 40279_2022_1784_MOESM1_ESM.pdf]

Title: Influence of Resistance Training Proximity-to-Failure on Skeletal Muscle Hypertrophy: A Systematic Review and Meta-Analysis.

Running title: Influence of Proximity-to-Failure on Muscle Hypertrophy.

Authors: Martin C. Refalo[1], Eric R. Helms[2], Eric. T. Trexler[3], D. Lee Hamilton[4], & Jackson J. Fyfe[4].

Affiliations:

1. Centre for Sport Research (CSR), School of Exercise and Nutrition Sciences, Deakin University, Geelong, Australia.
2. Sport Performance Research Institute New Zealand (SPRINZ), Auckland University of Technology, Auckland, New Zealand.
3. Trexler Fitness LLC, Raleigh, USA.
4. Institute for Physical Activity and Nutrition (IPAN), School of Exercise and Nutrition Sciences, Deakin University, Geelong, Australia.

Corresponding author:

Mr. Martin Refalo
Centre for Sport Research (CSR), School of Exercise and Nutrition Sciences
Deakin University, Geelong, Australia
Email: mrefalo@deakin.edu.au
ORCiD: 0000-0003-3755-6216

1. Sensitivity Analyses

1.1 Studies Comparing Set Failure VS. Non-Failure (Theme A and B)

Figure S1. Scatter Plot of P-Values Associated with r = 0.6 – 0.9

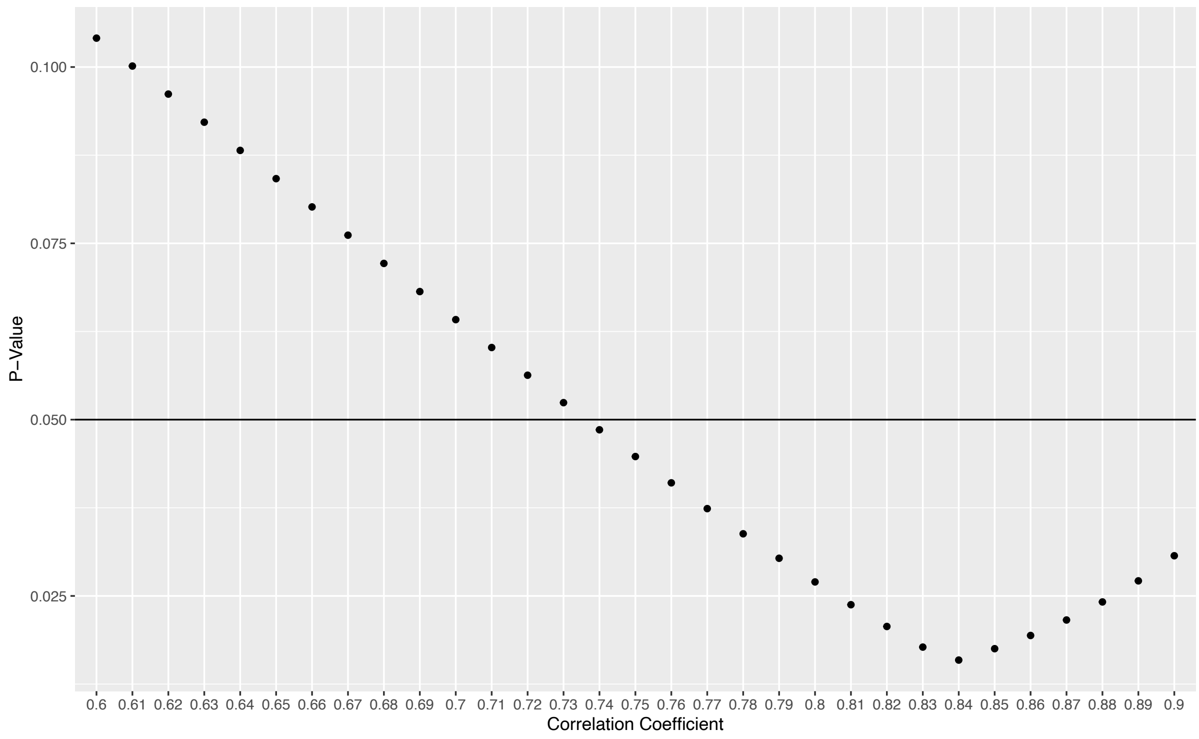

Figure S2. Scatter Plot of Effect Sizes Associated with r = 0.6 – 0.9

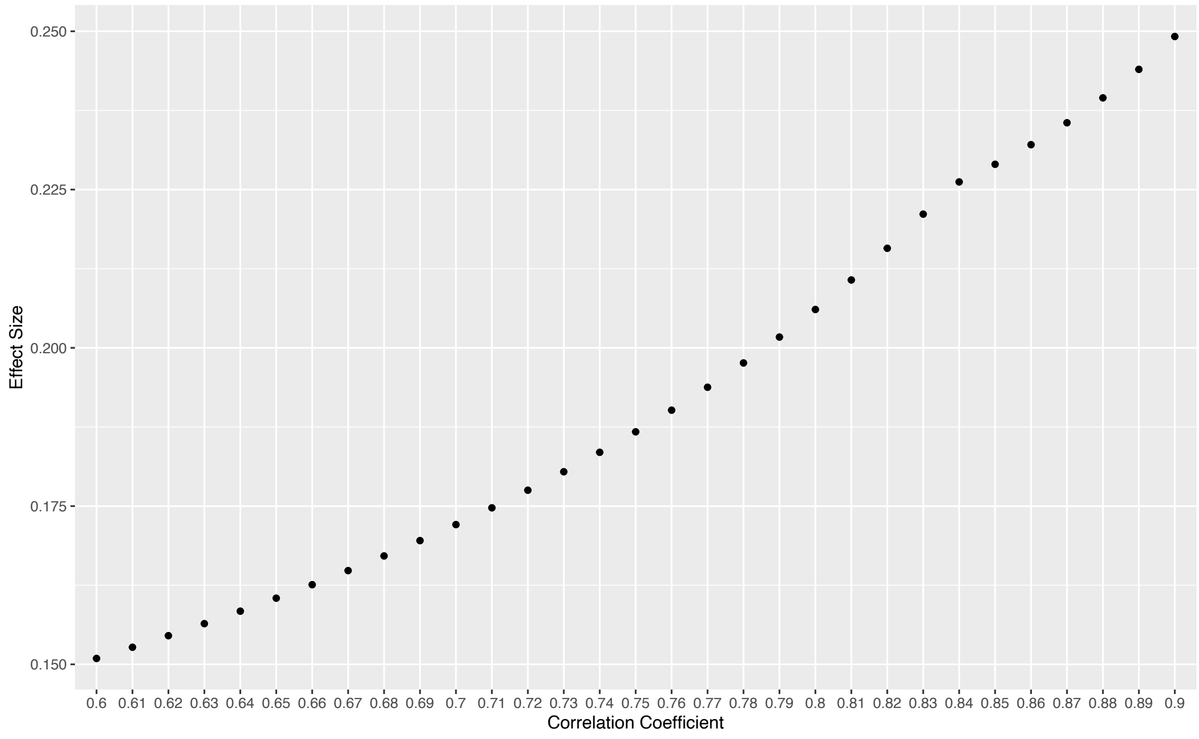

1.2 Studies Comparing High Velocity Loss VS. Moderate Velocity Loss (Theme C)

Figure S3. Scatter Plot of P-Values Associated with r = 0.6 – 0.9

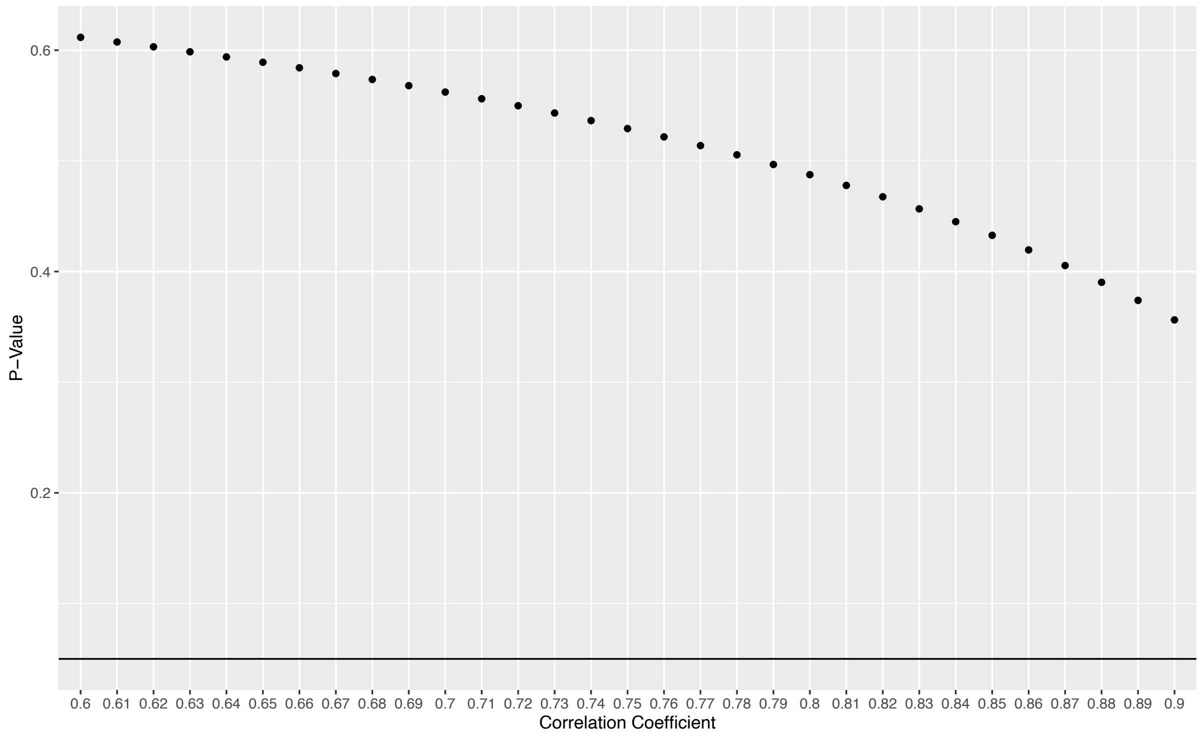

Figure S4. Scatter Plot of Effect Sizes Associated with r = 0.6 – 0.9

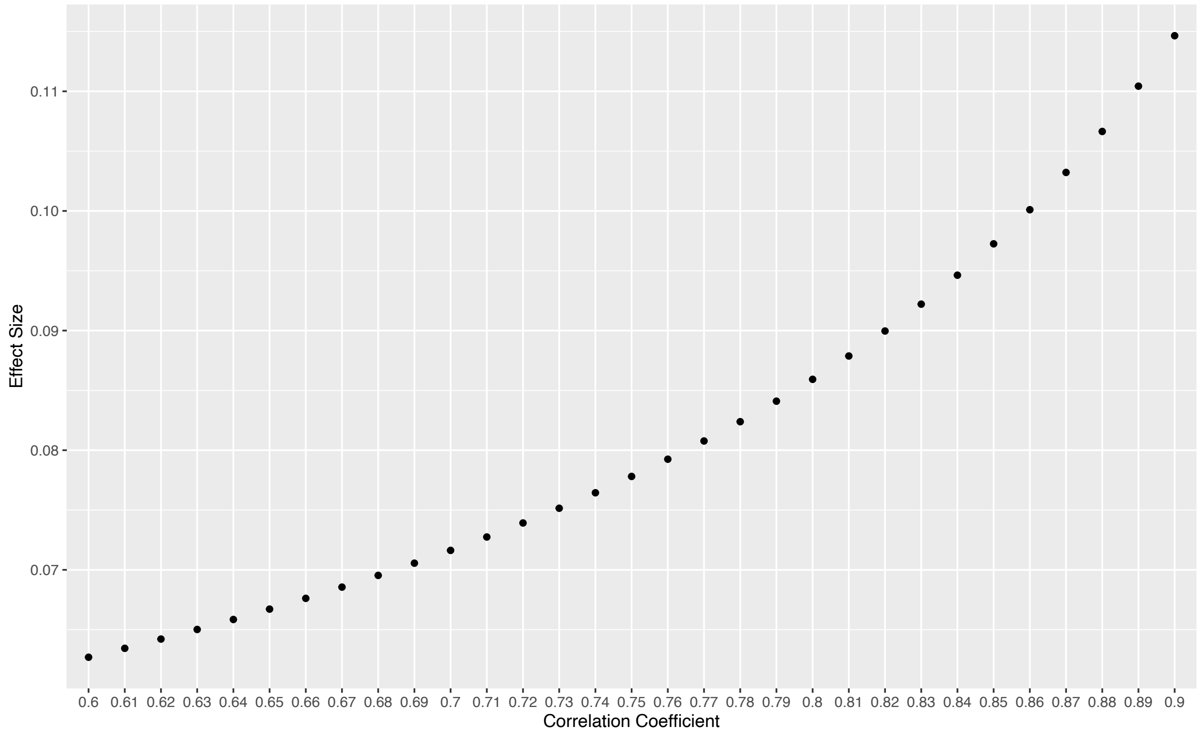

2. Publication Bias

2.1 Studies Comparing Set Failure VS. Non-Failure (Theme A and B)

Figure S5. Funnel Plot of All Effects for Studies in Theme A and B

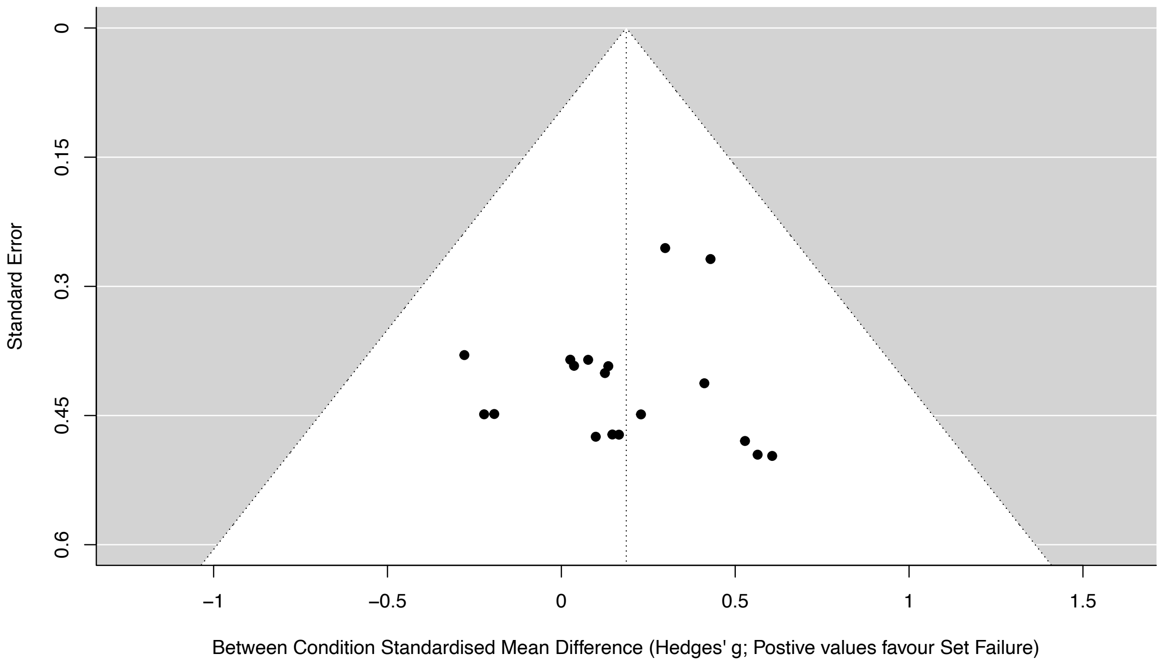

2.2 Studies Comparing High Velocity Loss VS. Moderate Velocity Loss (Theme C)

Figure S6. Funnel Plot of All Effects for Studies in Theme C

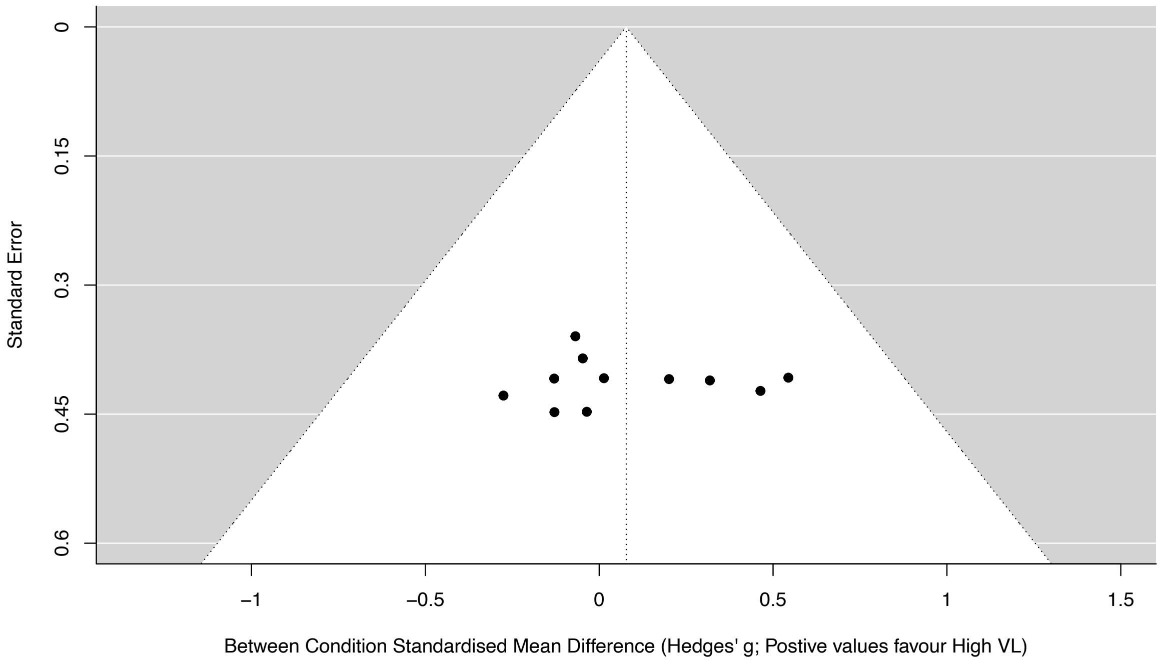

3. Methodological Quality Assessment

Table S1. Methodological quality for each included study assessed using the (TESTEX) scale.

**Study**	**TESTEX Scale Item**																
	**1**	**2**	**3**	**4**	**5a**	**5b**	**5c**	**6a**	**6b**	**6c**	**7**	**8**	**8**	**9**	**10**	**11**	**12**	**Total**
Andersen et al. 2021	1	1	0	1	No	No	0	0	1	1	0	1	1	1	0	1	1	10
Bergamasco et al. 2020	1	0	0	1	No	No	0	1	1	1	0	1	1	1	0	1	1	10
Karsten et al. 2021	1	0	0	1	No	No	0	1	1	1	0	1	1	1	0	1	1	10
Lacerda et al. 2020	1	1	0	1	No	No	1	1	1	1	0	1	1	1	0	1	1	12
Lasevicius et al. 2019	1	0	0	1	No	No	0	0	1	0	0	1	1	1	0	0	1	7
Martorelli et al. 2017	1	1	0	1	No	No	0	1	0	0	0	1	1	1	0	1	1	9
Nobrega et al. 2018	1	1	0	1	No	No	0	1	1	1	0	1	1	1	0	0	1	10
Pareja-Blanco et al. 2017	1	0	0	1	No	No	1	1	1	1	0	1	1	1	0	1	1	11
Pareja-Blanco et al. 2020a	1	0	0	1	No	No	1	0	1	0	1	1	1	1	0	1	1	10
Pareja-Blanco et al. 2020b	1	0	0	1	No	No	1	1	1	1	0	1	1	1	0	1	1	11
Rissanen et al. 2022	1	1	0	1	No	No	0	1	1	0	0	1	1	1	0	1	1	10
Rodiles-Guerrero et al. 2022	1	1	0	1	No	No	1	1	1	1	0	1	1	1	0	1	1	12
Sampson et al. 2020	1	1	0	1	No	No	0	1	1	1	0	1	1	1	0	1	1	11
Santanielo et al. 2020	1	0	0	1	No	No	1	0	1	1	0	1	1	1	0	0	1	9
Terada et al. 2021	1	0	0	1	No	No	0	1	1	0	0	1	1	1	0	0	1	8
